# Molecular Domino Toppling for Directed Self‐Erasing Information Transfer

**DOI:** 10.1002/adma.202419195

**Published:** 2025-04-07

**Authors:** Ying Li, Abhinav Chandresh, Hung‐Hsuan Lin, Nina Vankova, Dragos Mutruc, Thomas Heine, Stefan Hecht, Lars Heinke

**Affiliations:** ^1^ Karlsruhe Institute of Technology (KIT) Institute of Functional Interfaces (IFG) Hermann‐von‐Helmholtz‐Platz 1 76344 Eggenstein‐Leopoldshafen Germany; ^2^ Freie Universität Berlin Institute of Chemistry and Biochemistry Arnimallee 22 14195 Berlin Germany; ^3^ TU Dresden Fakultät für Chemie und Lebensmittelchemie, Bergstraße 66c 01062 Dresden Germany; ^4^ Humboldt‐Universität zu Berlin Department of Chemistry & Center for the Science of Materials Berlin Brook‐Taylor‐Strasse 2 12489 Berlin Germany; ^5^ Helmholtz‐Zentrum Dresden‐Rossendorf Forschungsstelle Leipzig Permoserstraße 15 04318 Leipzig Germany

**Keywords:** azobenzenes, metal–organic frameworks, molecular dominoes, smart material systems

## Abstract

In the pursuit of more secure information transfer, advanced nanoelectronic technologies and nanomaterials must be developed. Here, a material is presented able to undergo an unprecedented light‐pumped directional charge‐transfer process reminiscent of toppling dominoes. The material is based on *ortho*‐fluorinated azobenzene molecules which are organized in molecular rows by the regular array of a metal–organic framework. The azobenzene molecules undergo light‐induced *trans*→*cis* forward as well as electrocatalytic *cis*→*trans* backward isomerization. The findings reveal that electron hopping occurs in a sequential and propagating manner between the light‐generated *cis* isomers along with an isomerization of the sample to the *trans*‐state. Thus, light can be used to locally write information, which subsequently can be read out by the transferred charge with simultaneous deletion of the information. This freely repeatable, self‐erasing domino information transfer is a groundbreaking new mechanism to process information on the molecular level that may find application in encryption.

## Graphical abstract

An azobenzene‐based material with unprecedented light‐pumped directional charge‐transfer reminiscent of toppling dominoes is presented. After light‐induced *trans*→*cis* forward isomerization, electron hopping occurs in a sequential and propagating manner between the *cis* isomers along with an electrocatalytic *cis*→*trans* backward isomerization. This repeatable, self‐erasing domino information transfer is a groundbreaking new mechanism, suitable to process information on the molecular level.

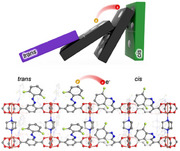

## 1 Introduction

Information storage and processing by functional organic molecules can possess advantages compared to the established device technologies based on inorganic semi‐conductors, including material price, flexibility, functionality, bio‐compatibility, and versatility.^[^ ^1^ ^]^ Molecular data storage is often based on photoswitchable molecules, which upon photon absorption reversibly interconvert between different (meta)stable switching states (ground state configurations).^[^ ^2^ ^]^ In that way, optical data storage with a high density of information (typically binary, i.e., ON‐OFF) has been realized.^[^ ^3^ ^]^ In addition, data processing using simple logic elements, such as AND, based on photoresponsive molecules has been demonstrated.^[^ ^4^ ^]^ For more advanced information storage and processing, we envision a system where the optical information writing is separated from an electronic read‐out, which is coupled with a simultaneous reset. Such a coupled system could be realized using domino‐toppling‐like directional information transfer.

Various domino or cascade processes were established for different chemical reactions involving various classes of molecules.^[^ ^5^ ^]^ Furthermore, repeatable cascade reactions in solution based on photoswitchable molecules were also explored.^[^ ^6^ ^]^ There, the information is passed on from one compound to the next one. This means, the output of one molecular logic unit serves as the input for another (different) molecular logic unit.^[^ ^6a–c^ ^]^ So far, these reactions take place randomly in the liquid phase and have not been achieved in the solid‐state – a prerequisite for practical devices. Moreover, the realization of ordered arrays allowing for directional and repeatable information transfer remains an unsolved challenge.

Azobenzene is a widely used photoswitchable molecule, which can isomerize between its *trans* (*E*) and *cis* (*Z*) forms.^[^ ^7^ ^]^ The isomerization from the thermodynamically stable *trans* form to the *cis* form is powered by photoexcitation. The isomerization of the *cis* back to the *trans* form can occur upon excitation with light of a different wavelength or by (typically slow) thermal relaxation in the dark. By functionalizing azobenzene with *ortho*‐fluorine substituents, the *trans*→*cis* isomerization can be performed by irradiation with green‐light of 530 nm and the *cis*→*trans* isomerization can be performed by irradiation with violet‐light of 400 nm.^[^ ^8^ ^]^ Both isomerization paths are based on the excitation of the n‐π^*^ transition with visible‐light. For the *ortho*‐fluorinated azobenzene chromophores, the thermal relaxation of the *cis* isomers back to the thermodynamically stable *trans* isomers is rather slow, with a half‐life of up to 2 years at room‐temperature.^[^ ^8^, ^9^ ^]^ However, it has been shown that the *cis*→*trans* relaxation process of azobenzene molecules in solution can be electrocatalytically accelerated, i.e., by electron/hole catalysis.^[^ ^2^, ^10^ ^]^ Specifically, when the initial *cis* azobenzene is reduced, the activation barrier for the *cis*→*trans* relaxation of the radical anion is significantly smaller.^[^ ^10a^ ^]^ This results in a fast and quantitative isomerization back to the *trans* isomer. The reduction and oxidation have been used to efficiently isomerize various functional molecules.^[^ ^6e,f^ ^]^ It has been shown that the electron transfer between the *trans* azobenzene radical anion and another neutral *cis* azobenzene can result in chain reactions where the photon quantum yield is larger than 100%.^[^ ^6d,f^ ^]^ To date, information transfer using such chain reactions has not yet been realized.

The development of photoresponsive materials as promising key component in devices represents a grand challenge in material science.^[^ ^11^ ^]^ Often, photochromic molecules are incorporated in polymers, resulting in photoswitchable amorphous materials.^[^ ^12^ ^]^ For many applications, however, the immobilization of the photoswitches in an organized array, which confines the individual isomerization events yet leaves sufficient space, is desired. This can be realized using metal–organic frameworks (MOFs), a class of crystalline nanoporous materials composed of metal nodes which are connected by organic linker molecules.^[^ ^13^ ^]^ In recent years, various MOFs with incorporated photoswitches have been presented.^[^ ^14^ ^]^ Such MOFs either in the form of powders or in the form of thin films were used to demonstrate the light‐induced remote‐control of various functionalities, such as photoswitchable adsorption, diffusion, membrane permeation, and proton conduction.^[^ ^15^ ^]^ To date, the multi‐stimuli response of such materials has not yet been explored.

Here, we disclose multi‐stimuli responsive MOF thin films composed of *ortho*‐fluorinated azobenzene units organized in a periodic array, in which the *trans*→*cis* isomerization can be induced by visible‐light absorption and thermal *cis*→*trans* back isomerization is accelerated by applying an electric field. The latter induces an electrocatalytic reversion process back to the *trans*‐state, proceeding along with a domino‐toppling‐like directional transport of an electron, thereby coupling both, the read‐out and erasing, processes. In these material systems, information is written by light, while an electric current is used for retrieving and deleting the information at the same time. Thus, for the first time, a material allowing for repeatable optoelectronic information storage and self‐erasing read‐out could be realized. This is the molecular nanotechnology realization of an automatically self‐destructed message after one‐time reading, just as in the “Mission Impossible” movies or in the cartoons of Inspector Gadget.

## 2 Results and Discussion

**Figure 1:**
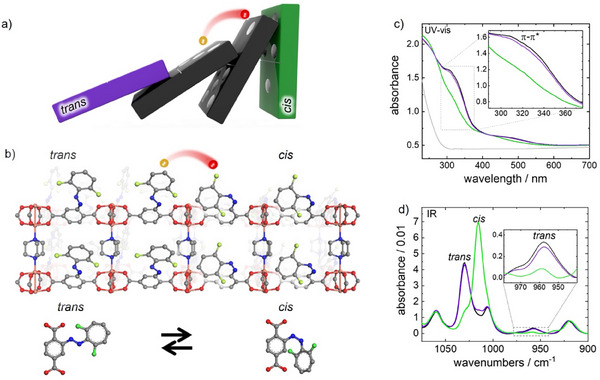
a) Concept of information transfer sketched as an analogy to a domino chain: The electron transfer (red sphere, from left to right) results in consecutive toppling of the upright dominoes, symbolizing the meta‐stable *cis* isomers which convert to the stable *trans* isomers, i.e., lying dominoes. b) The structure of the Cu_2_(F_2_AzoBDC)_2_(dabco) SURMOF film. Sketch of the overturning of the *cis* isomers (right) to the *trans* isomers (left). The *trans* and *cis* isomers of individual F_2_AzoBDC molecules are shown below. Color code: C (gray), O (red), N (blue), Cu (orange), and F (green). H not shown. c) UV–vis transmission absorbance spectra and d) infrared reflection absorption spectra (IRRAS) of Cu_2_(F_2_AzoBDC)_2_(dabco) SURMOF. The sample was in the thermally relaxed state (with all azobenzene moieties in the *trans*‐state, black), upon irradiation with green‐light (resulting in the *cis‐*rich state, green), and upon irradiation with violet‐light (resulting in the *trans*‐rich state, violet). The gray line in panel c) is the UV–vis spectrum of the IDE@glass substrate, on which the SURMOF was grown. The insets show magnifications of the *π–π* ^*^ *trans* azobenzene band in c) and of an isolated *trans* azobenzene vibrational IR band in d).

The structure of the MOF film with the azobenzene side groups in their *trans* and *cis* configurations is shown in **Figures** **1**b and S1 (Supporting Information). The MOF has a pillared‐layer structure, referred to as Cu_2_(F_2_AzoBDC)_2_(dabco), where dabco stands for 1,4‐diazabicyclo[2.2.2]octane and F_2_AzoBDC for (*E*)‐2‐((2,6‐difluorophenyl)diazenyl)terephthalate. The MOF films were prepared in a layer‐by‐layer fashion directly on the substrates, resulting in surface‐mounted metal–organic frameworks, referred to as SURMOFs.^[^ ^16^ ^]^ The crystallinity of the films was explored by X‐ray diffraction (XRD) in in‐plane and out‐of‐plane geometry (Figure S1, Supporting Information). The XRD data show that the film is crystalline and the film was grown in an oriented fashion with the [001] direction perpendicular to the substrate surface. The scanning electron microscopy (SEM) images of samples grown on glass substrates with deposited interdigitated gold electrodes (IDE, Figure S2, Supporting Information) show that the SURMOF homogeneously covers the substrate surface. The film is composed of intergrown crystallites and the film thickness is ≈0.2 µm.

UV–vis spectroscopy in transmission mode was used to explore the *trans*→*cis* photoswitching, where the sample was irradiated with green‐light of 530 nm and violet‐light of 400 nm. The UV–vis spectra, Figure 1c, show reversible changes of the intensity of the *π–π* ^*^ as well as *n*‐*π* ^*^ transitions of the azobenzene ≈320 and 450 nm, respectively. These changes are a clear indication for the green‐light induced *trans*→*cis* isomerization and violet‐light induced *cis*→*trans* isomerization, in agreement with previous studies.^[^ ^17^ ^]^

Infrared spectroscopy, precisely infrared reflection–absorption spectroscopy (IRRAS), was employed to quantify the ratio of both azobenzene isomers in the SURMOF (Figure 1d). In the spectra, the bands related to the SURMOF scaffold remained unchanged upon light irradiation, whereas the vibrations of the fluorinated azobenzene side group changed significantly. Upon green‐light irradiation, the band at 1015 cm^−1^, which is attributed to the *cis* isomer, increases. At the same time, the intensity of the band at 960 cm^−1^, attributed to the *trans* isomer,^[^ ^17b^ ^]^ decreases to 14% of the intensity of the thermally relaxed 100%‐*trans* SURMOF. Thus, a *cis* ratio of 86% was obtained. Upon violet‐light irradiation, the *cis* bands decrease while the *trans* bands increase again, giving rise to 87% recovery of the initial *trans* content (based on the intensity change of the band at 960 cm^−1^).

**Figure 2:**
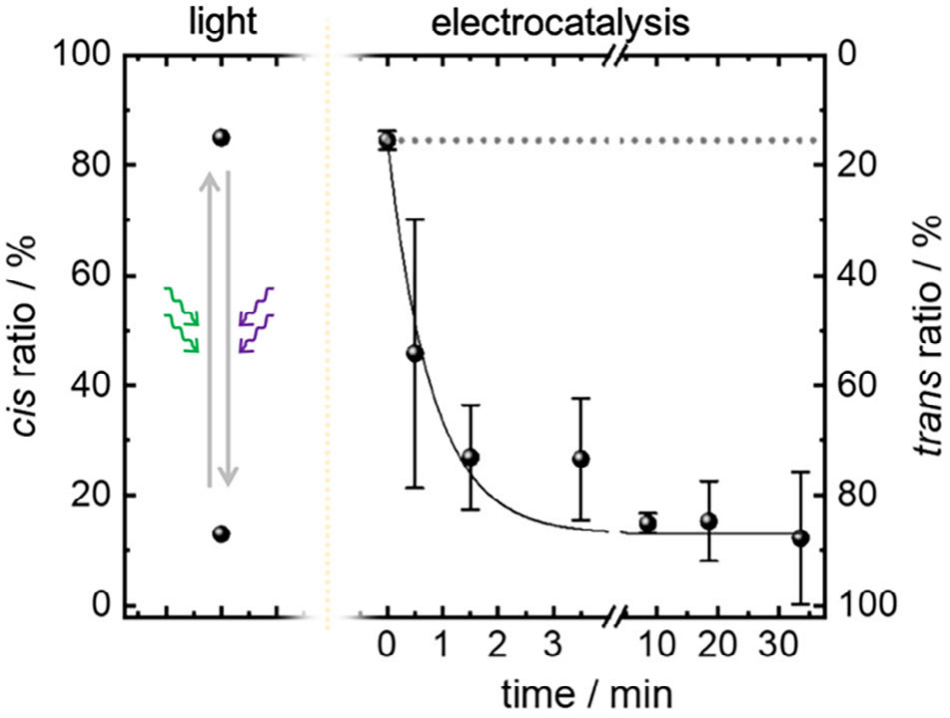
*trans‐cis* isomerization of azobenzene by light (left) and electrocatalytically (right, without light). By light, the *cis*‐rich state is obtained by green‐light irradiation, and the *trans*‐rich state is obtained by violet‐light irradiation. For the electrocatalytical *cis*→*trans* isomerization, a voltage of 10 V was applied to the *cis*‐rich SURMOF sample, which was obtained by green‐light irradiation. The isomer ratios were calculated from UV–vis spectra (Figure S4, Supporting Information), and the average of four runs with the standard deviation as error bars are shown. The solid black line is a mono‐exponential decay function with a time constant of 0.8 min, describing the data. For better visibility, the scaling of the *x*‐axis changes after 4 min. In comparison, the thermal relaxation in the dark without an electric field (Figure S3, Supporting Information) is shown as a dotted gray line, which is virtually horizontal.

Then, the electrocatalytic *cis*→*trans* switching of the azobenzene groups in the SURMOF was explored. For this purpose, the SURMOF films were prepared on glass substrates with two deposited interdigitated gold electrodes (IDEs), between which an electric field can be applied. This is in contrast to previous studies of electrocatalytic *trans*→*cis*‐isomerization, where electrochemical three‐electrode setups with (liquid) electrolytes were used.^[^ ^2^, ^10^ ^]^ The yield of the *trans* and *cis* azobenzene in the SURMOF was calculated by their relative absorbance at 320 nm (*π–π* ^*^ *trans* azobenzene) in the UV–vis spectra, which were measured in transmission through the SURMOF@IDE@glass‐substrate sample. The amount of *trans* and *cis* azobenzene was calibrated by the IR data, showing 13% and 86% *cis* for the sample upon violet‐ and green‐light irradiation (see above). By applying an electric field between the electrodes of the IDE@glass substrate, the amount of *cis* azobenzene in the SURMOF decreased and the *trans* amount increased (**Figure** **2**). After ≈3–10 min, the final *trans*‐rich state was reached. The conversion from *cis* to *trans* azobenzene can be described with a mono‐exponential decay function with a time constant of ≈0.8 min (Figure 2). Importantly, this time constant for the *cis*→*trans* isomerization is ≈1 million times faster than the thermal relaxation in the dark without applying an electric field at room‐temperature (≈1.6 years, see Figure S3, Supporting Information).

The process can be reliably repeated for many cycles without significant fatigue (Figure S4, Supporting Information). Please note, that the XRD data show that the crystallinity was not affected by the *trans*‐*cis* cycles caused by illumination and the electric field (Figure S1, Supporting Information) and therefore, the applied wavelength and voltage do not harm the structure of the SURMOF film. For a second sample, a similar electric field‐induced *cis*→*trans* isomerization was observed (Figure S5, Supporting Information). Compared with smaller voltages, such as 1 and 5 V, the voltage of 10 V, corresponding to an electric field of 1 V µm^−1^, shows the most effective performance (Figure S6, Supporting Information).

**Figure 3:**
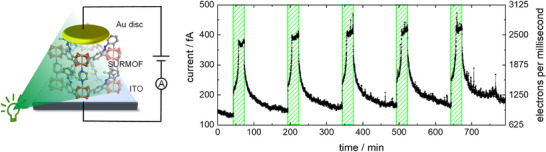
Current through SURMOF film upon repeated exposure to green‐light followed by no irradiation. The setup consists of ITO (on glass) as the bottom electrode and a small gold disc as the top electrode (left), applying a constant (DC) voltage of 1 V. The sample was irradiated five times for 30 min with a green‐light of 530 nm (green‐shaded boxes). Initially, the sample was in the *trans‐*state and equilibrated at a voltage of 1 V for 1 h.

Next, the electrocatalytic *trans*→*cis* switching was used for demonstrating information transfer. To this end, the SURMOF was grown on transparent and conducting indium‐tin‐oxide (ITO) substrates, which allow for illumination from below. On top of the SURMOF, a small gold disc was used as the top electrode,^[^ ^18^ ^]^ and a DC voltage of 1 V was applied between the ITO bottom electrode and the gold top electrode. While the current was recorded (**Figure** **3**), the sample was irradiated with green‐light, converting the azobenzene units from the *trans* to the *cis* isomer. Formation of the latter caused an initial increase of the current from ≈150 to 400 fA. Upon switching off the light, the current decreased back to the base current (≈150 fA) within a time constant of ≈10 min. This process can be repeated several times. Upon irradiation with red‐light of similar intensity (Figure S7, Supporting Information), no significant current increase was observed, indicating that the observed current increase is not due to thermal effects. Moreover, the time scales of current increase and decrease indicate that the phenomenon is not caused by plain photoconduction,^[^ ^19^ ^]^ in which case the current would increase/decrease instantly (within ⪅1 s). Repeating the experiments with a different sample (Figure S7, Supporting Information) provided essentially the same results.

Considering the base current of ≈150 fA in combination with the electrode surface area (1.25 mm^2^, assuming perfect contact) and the film thickness (1.6 µm) gives a conductivity of ≈2 10^−15^ S m^−1^. This very small conductivity indicates the highly insulating character of the MOF and that charge‐transfer through the MOF backbone, i.e., along the Cu‐paddle wheels connected by the dabco pillars, is strongly suppressed.

The current change of ≈250 fA, which corresponds to ≈1500 electrons per millisecond, decays with an average time constant of 15.5 ± 4.8 min (average value with standard deviation of the mono‐exponential decays in Figure 3). This decay is somewhat slower than the one observed previously in Figure 2, most likely due to the smaller applied voltage, i.e., 1 V versus 10 V. From the current and the time constant, an average transferred charge of 0.23 nC follows. Considering the size of the top electrode (1.25 mm^2^) and assuming perfect and homogeneous contact to the SURMOF film, this charge indicates that 0.0013 electrons were transferred through each unit cell (1.06 nm x 1.06 nm) during the process. In addition to defects that trap the electrons, the main reason for the low electron transfer efficiency as compared to the perfect system where one electron per unit cell would be transferred (see below) appears to be the *trans*→*cis* conversion which is significantly smaller than 100%. Please note that the (unusual) shape of the current curve is straightforwardly qualitatively reproduced by simple Monte‐Carlo simulations of the domino‐like charge‐transfer, Figure S10 (Supporting Information).

**Figure 4:**
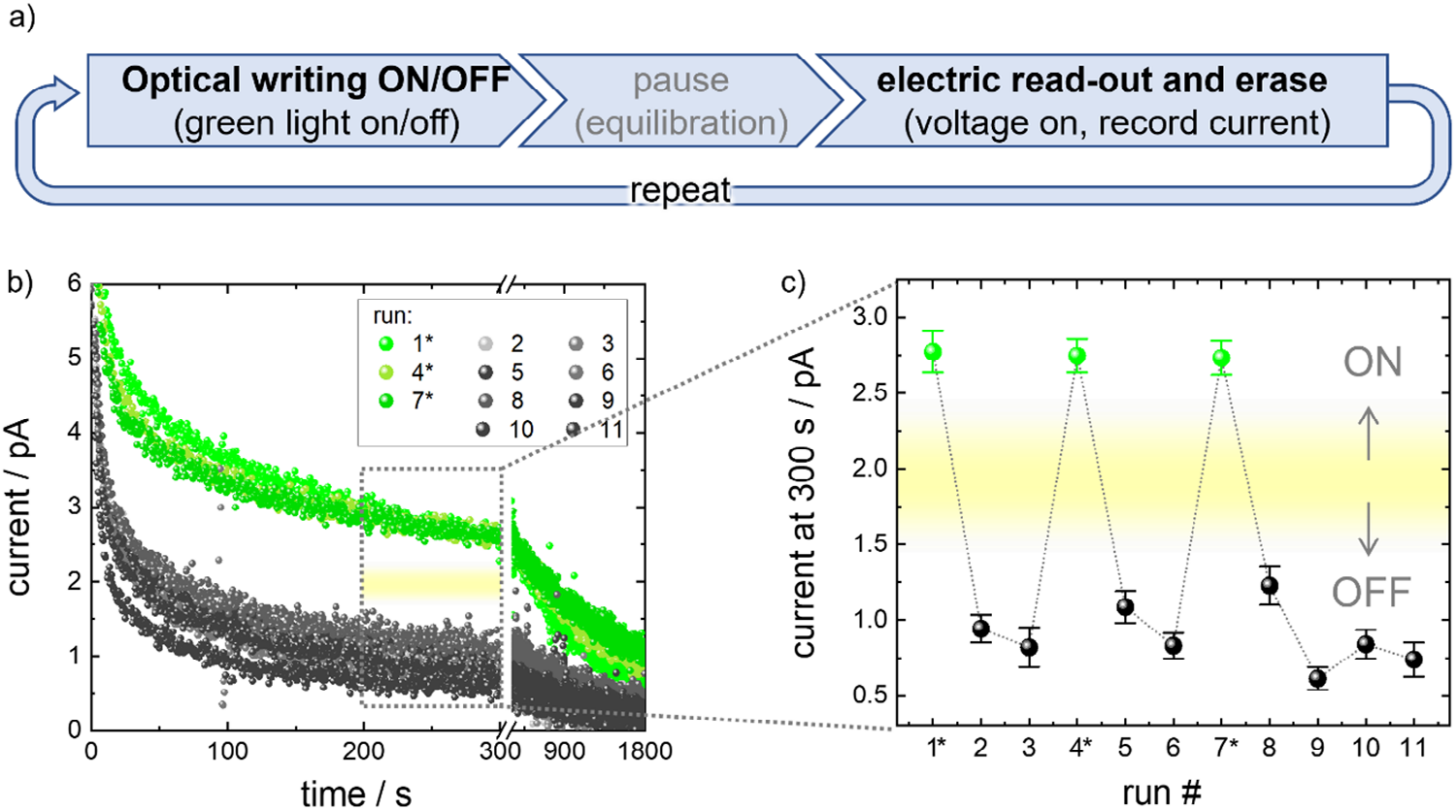
Self‐erasing current read‐out. a) Sketch of the optical writing and electric read‐out with coupled information erasing. At the beginning of each cycle, the information is written by a green‐light. Here, in runs 1, 4, and 7 (labeled by asterisks and highlighted in green), the sample was irradiated with green‐light, causing *trans*→*cis* switching in the SURMOF (ON‐state), otherwise the sample remains in the *trans* (OFF) state. After equilibrating the sample without applying a voltage for 30 min, 1 V DC voltage was switched on and the current was measured for 30 min. Then, 11 such cycles using the setup depicted in Figure 3 were performed. b) Transient current through the SURMOF film. c) The current averaged over a time range of 200–300 s, labeled by the dotted frame in b), for the 11 cycles. The error bars are the standard deviations of the current during the time interval. The yellow bars in b) and c) mark the threshold between the ON state (initially *cis*) and the OFF state (initially *trans*).

The current can be used as the signal of the system allowing one‐time, self‐erasing read‐out of the *trans*/*cis* information written prior by light. To demonstrate this, the SURMOF film on the ITO substrate with the gold top electrode was prepared in either the *cis*‐rich or in the *trans*‐rich state, by illuminating with green‐light or leaving it in the dark. Then, the voltage between the two electrodes was switched on (**Figure** **4**). In all cases, when switching on the voltage, a relatively large current of ≈6–10 pA was observed, which we attribute to the charging of the capacitor composed of the two electrodes with the SURMOF as dielectric in between (see Figure 3). Remarkably, for the SURMOF initially in the *cis* state, the current was significantly higher than the current of the sample in the *trans*‐state. The ratio in the current of both switching states is approximately three. After 30 min, in all cases – regardless of if the SURMOF was initially in the *trans* or *cis* state – a final current of less than 1 pA was reached. The small yet recurring current indicates that the initially *cis*‐configured azobenzene moieties in the SURMOF were converted back to their *trans* form. Hence, and very importantly, after the read‐out by the current, the sample is always in the *trans*‐state and the optically written *trans/cis* information in the SURMOF is deleted.

**Figure 5:**
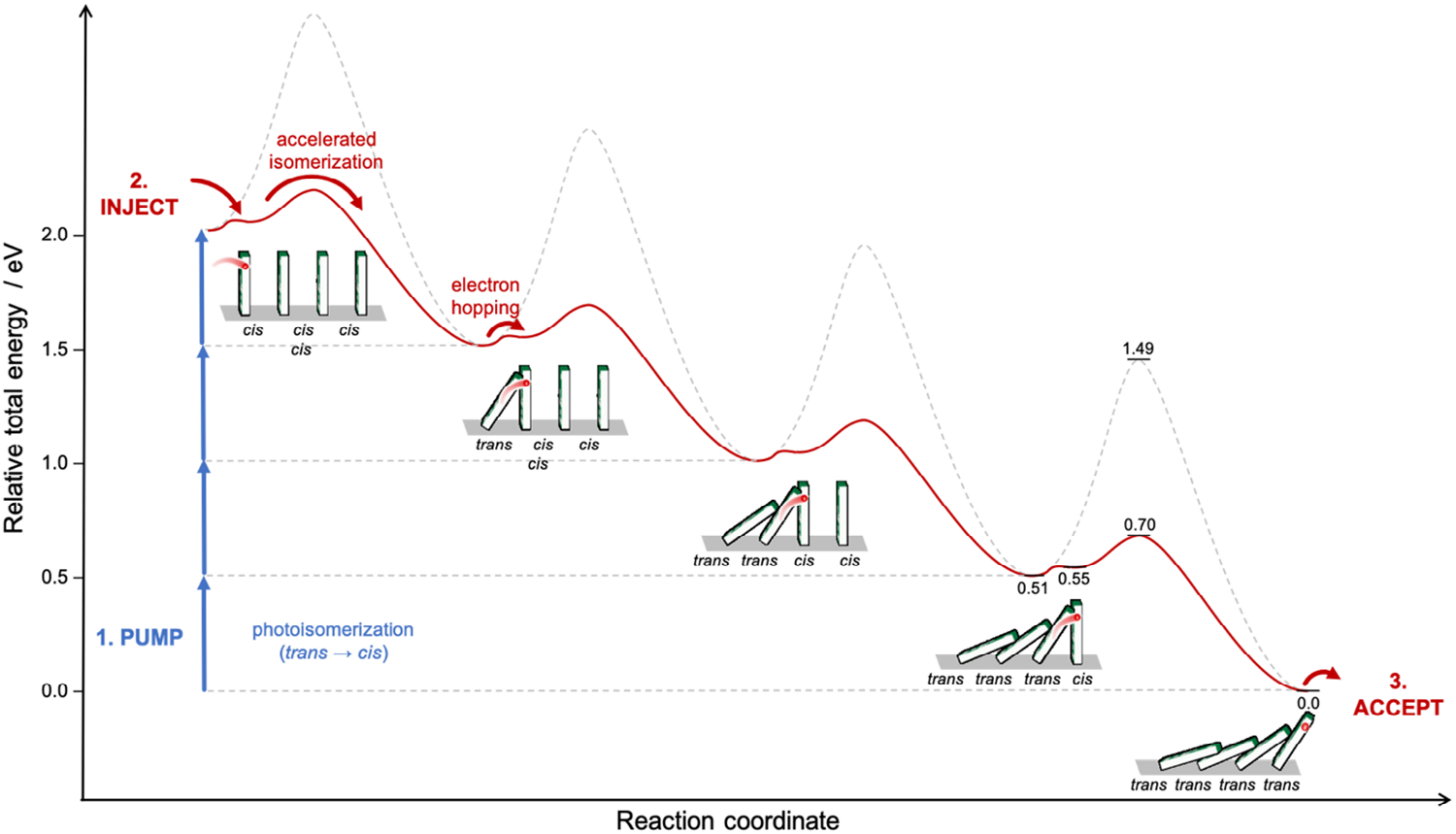
Mechanism of light‐pumped directional electron transport in azobenzene SURMOF material illustrated for a tetrameric unit (dominos symbolize individual azobenzene units): Initial *trans*→*cis* photoisomerization (“pumping,” blue) gives kinetically stable all‐*cis* state (top left). Subsequent electron transfer to the first *cis* isomer in the row induces fast thermal *cis*→*trans* isomerization followed by electron transfer to the neighboring *cis* isomer to carry on the chain reaction and transporting the electron to a trap site while resetting the system to its all‐*trans* resting state (bottom right). Energies of minima and transition states were computed in the gas phase at the B3LYP‐D3(BJ)/TZ2P level of theory with ADF2019 and are drawn to scale with relevant energy values labeled for the final 4th isomerization event (for further details see Figure S9, Supporting Information). For comparison, barriers for thermal *cis*→*trans* isomerization of neutral azobenzene are shown in gray dotted lines.

For a better understanding of the *cis*→*trans* switching and charge‐transfer mechanism of the azobenzene groups in the SURMOF, we performed density functional theory (DFT) calculations considering the isolated *cis* and *trans* isomers of the F_2_AzoBDC molecule in the gas phase. As expected, *trans*‐F_2_AzoBDC was found to be the lowest energy isomer, supporting the experimental characterization of the synthesized SURMOF with azobenzene linkers in the *trans* configuration only. According to the calculations, the *trans*‐F_2_AzoBDC molecule is 0.51 eV more stable than the *cis* isomer. The latter, however, is kinetically stable, with a thermal barrier of 0.98 eV (at the B3LYP‐D3(BJ) level of theory) preventing its back isomerization to the *trans* isomer (upper reaction path in Figure S9, Supporting Information). Comparing the energies of the corresponding singly reduced species, i.e., the radical anions *trans^•^* ^−^ and *cis^•^* ^−^, shows a similarly large thermodynamic driving force of −0.55 eV for *cis^•^* ^−^→*trans^•^* ^−^ isomerization but in combination with a largely reduced thermal barrier of only 0.15 eV (lower reaction path in Figure S9, Supporting Information). To evaluate the feasibility of an electrocatalytic *cis*→*trans* isomerization process, we considered an adiabatic process and computed that the passing on of one electron from a reduced *trans^•^* ^−^ to a neutral *cis* isomer is slightly uphill by + 0.038 eV. Since such hopping, however, will immediately result in a successive irreversible *cis^•^* ^−^→*trans^•^* ^−^ isomerization event with a significant energy gain of −0.55 eV (see above), the overall process is highly exergonic (+0.038 + −0.55 = −0.51 eV per azobenzene unit) and thus should lead to efficient and complete *cis*→*trans* switching of the entire SURMOF. To illustrate the overall process, we show the sequence of alternating electron transfer and *cis*→*trans* isomerization events in an array of four neighboring azobenzene units (**Figure** **5**).

After optically pumping the initial, thermally relaxed 100%‐*trans* SURMOF to an all‐*cis* state, reduction at the electrode (i.e., the cathode) injects electrons into a *cis*‐configured azobenzene unit, which rapidly isomerizes into its more stable *trans* form and subsequently transfers the electron to a nearby *cis* isomer. Repetition of these events in a propagating domino cascade leads to fast and complete isomerization of the entire system. As a result, an electron is directionally translocated from the initial injection to the final trap site (i.e., the anode) and the material is reset to the initial state (thermodynamically‐stable all‐*trans*, i.e., fallen dominoes). It is important to note that the thermal relaxation time of the (meta‐stable) *cis* azobenzene units in the dark without an electric field is ≈2 years, resulting in a rather stable and thus non‐volatile information transfer material system.

In the experimental setup, the electron injection occurs only at the cathode. At the anode, the electrons leave the system again. Thus, the direction of the domino toppling is from the cathode to the anode, which is also the direction of the force on the electrons in the electric field. In the present study, we focus on the charge‐transfer by single‐electron processes. In principle, higher excitations and multi‐electron processes cannot be excluded. However, such processes are very unlikely, especially with respect to the very small currents.

## 3 Conclusion

A multi‐stimuli responsive material was developed by organizing azobenzene molecules into an ordered array using metal–organic framework thin films. The azobenzene molecules undergo light‐induced *trans*→*cis* and *cis*→*trans* isomerization as well as electric‐field‐induced *cis*→*trans* isomerization in combination with electron transfer between adjacent azobenzene units in the regular array. Taking advantage of both stimuli, the azobenzene molecules are initially pumped by light to the *cis* state, in which the information is written. The information is subsequently read out by an electric field using the current as signal. Charge‐transfer occurs in a directed domino‐like chain where the azobenzene units are converted from the metastable *cis* to their stable *trans* isomers and an electron is passed along to the next unit. As a result, a current is flowing and the information, i.e., the *cis* state, is simultaneously deleted. Our system is the first demonstration of a cooperative domino‐toppling‐like information transfer in a molecular system. We envision that such concepts and derived materials will find application in data storage and encryption. Apart from azobenzene in MOFs, the concept should be widely applicable to other classes of photoswitchable molecules and materials ranging from highly ordered covalent‐organic frameworks to flexible single polymer chains decorated with proper functional side groups.

## 4 Experimental Section

### SURMOF Synthesis

The thin films of metal–organic framework with Cu_2_(F_2_AzoBDC)_2_(dabco) structure was synthesized in a layer‐by‐layer (lbl) fashion, resulting in surface‐mounted metal–organic frameworks (SURMOFs).^[^ ^16^ ^]^ In detail, the Cu_2_(F_2_AzoBDC)_2_(dabco) thin films were prepared by dipping the substrate in ethanolic solution of the MOF components, which were the metal nodes (copper acetate with a concentration of 1 mm for 5 min) and the linker molecules (F_2_AzoBDC and dabco with a concentration of 0.2 mm each for 25 min). The abbreviations are F_2_AzoBDC for (*E*)‐2‐((2,6‐difluorophenyl)diazenyl)terephthalic acid and dabco for 1,4‐diazabicyclo[2.2.2]octane. While F_2_AzoBDC was synthesized as described in ref. [17, 20], the other MOF components were purchased from Merck/Sigma–Aldrich. The substrates of the SURMOF films were interdigitated gold electrodes (IDEs) on glass sheets, which were obtained from Metrohm/NanoSPR. The total length of the gap between the gold electrodes was 1.68 m and the gap width was 10 µm. The ITO on quartz glass substrates were purchased from Sigma–Aldrich. The gold discs had a diameter of 1.26 mm diameter and a thickness of 10 µm. The gold discs were gold preforms purchased from AIM solder, Canada. Before the SURMOF preparation, the IDE@glass and ITO substrates were cleaned and functionalized with UV/Ozone for 15 min. The gold substrates were functionalized by immersion in 11‐mercaptoundecanol self‐assembled monolayer (MUD SAM). The lbl‐SURMOF‐synthesis process was repeated for 50 cycles for the films on the IDE@glass substrates (for UV–vis) and for the films on the gold substrates (for IRRAS) and for 250 cycles for the samples on the ITO and gold substrates (for the current measurements).

### Characterization

X‐ray diffraction measurements in out‐of‐plane geometry were carried out using a *Bruker D8 Advance* diffractometer equipped with a position‐sensitive detector in *θ–θ* geometry. The in‐plane XRD data were measured using a *Bruker D8 Discover* diffractometer, equipped additionally with a tilt‐stage, a quarter Eulerian cradle, and 2.3° Sollar‐slits. For out‐of‐plane and in‐plane XRD, a Cu‐anode with an X‐ray wavelength of λ = 0.154 nm was used.

Fourier‐transform infrared reflection absorption spectrometer (IRRAS) Bruker Vertex 80 was used for the vibrational spectroscopy of the samples. The spectra were recorded in grazing incidence reflection mode at an angle of incidence of 80° relative to the surface normal using a liquid nitrogen‐cooled mercury–cadmium–telluride mid‐band detector.

UV–vis absorption spectra of the SURMOF sample on the IDE@glass‐substrate were recorded with an Agilent Cary 5000 UV–vis–NIR spectrophotometer in transmission mode. To realize the 100%‐*trans*‐state before the UV–vis experiments, the sample was thermally relaxed at 70‐80 °C for 72 h. The electrocatalytic *cis*→*trans* isomerization was induced by applying an electric field of 1 V µm^−1^. This was realized by applying a constant DC voltage of 10 V at the IDEs. The voltage was applied for 0.5, 1.5, 3.5, 8.5, 18.5, and 33.5 min, respectively. The experiments were performed in pure nitrogen, avoiding water and other guest molecules in the pores.

The light irradiation was performed with LEDs from PrizMatix and using light‐cables. The intensities of the light irradiation are ≈3 mW cm^−2^ (400 nm), 2 mW cm^−2^ (530 nm), and 6 mW cm^−2^ (640 nm).

### Computational Details

All calculations were performed using the Amsterdam Density Functional (ADF 2019).^[^ ^21^ ^]^ The neutral and anionic *trans* and *cis* isomers of the F_2_AzoBDC molecule were optimized at the DFT level in the gas phase, using the dispersion‐corrected B3LYP‐D3(BJ) hybrid functional^[^ ^22^ ^]^ and Slater‐type all‐electron basis sets of TZ2P quality.^[^ ^23^ ^]^ Linear transit (LT) calculations were performed at the same level of theory to obtain an approximate path over the transition states between the *trans* and *cis* forms (neutral as well as anionic) of F_2_AzoBDC. To estimate the electron hopping energetics, we assumed that charge‐transfer is much slower than structural relaxation. The energy barrier associated with the transfer of an electron from *trans^−^* to *cis* F_2_AzoBDC linker was calculated as the difference between the ionization potential (IP) and the electron affinity (EA) of the respective local minimum structures.

## Conflict of Interest

The authors declare no conflict of interest.

## Supporting information



Supporting Information

## Data Availability

The data that support the findings of this study are available in the Refubium repository under https://doi.org/10.17169/refubium‐46820.
