# Supplementary material for: Molecular Domino Toppling for Directed Self‐Erasing Information Transfer
Source: Adv Mater. 2025 Apr 7;37(26):2419195. doi: 10.1002/adma.202419195 (PMC12232227; doi:10.1002/adma.202419195)
Supplement: Supplementary file 1 — Supporting Information [file ADMA-37-2419195-s001.docx]

Supporting Information

Molecular domino toppling for directed self-erasing information transfer

Ying Li,^1^ Abhinav Chandresh,^1,5^ Hung-Hsuan Lin,^2^ Nina Vankova,^2^ Dragos Mutruc,^3^ Thomas Heine,^2,4^ Stefan Hecht^3,*^ and Lars Heinke^1,5,*^


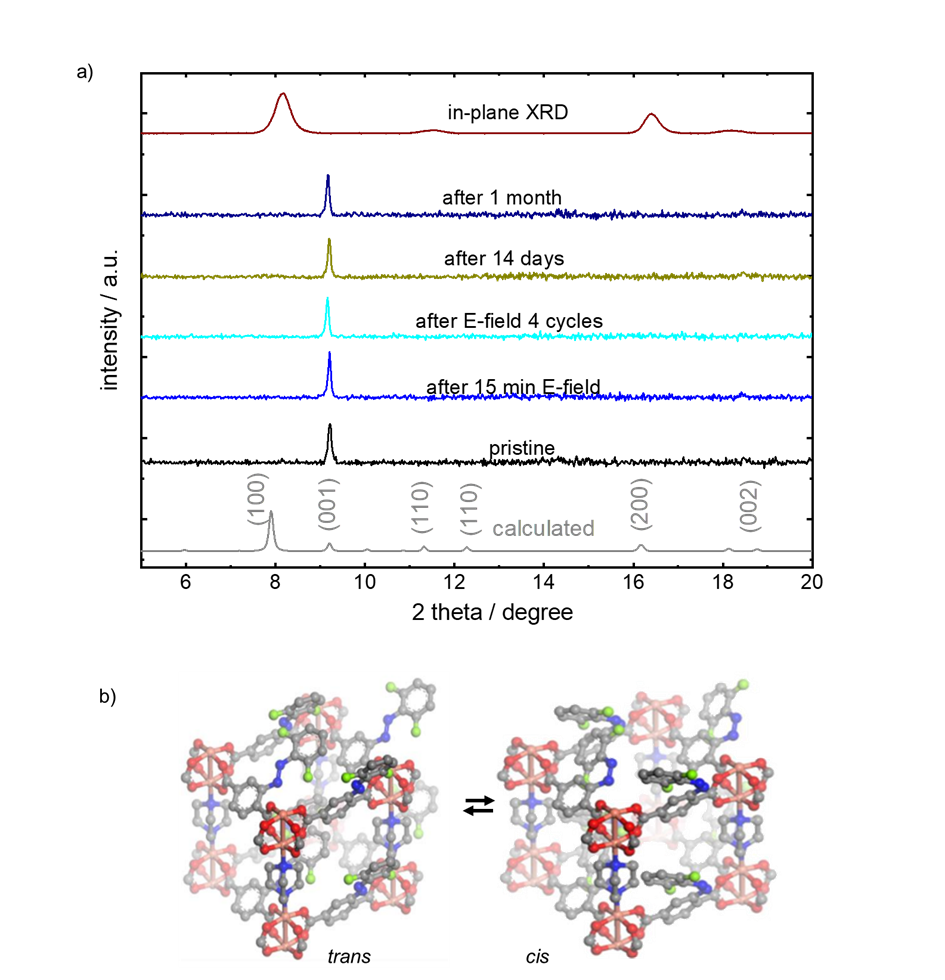


**Figure S1**. **a)** Out-of-plane X-ray diffractogram of the pristine Cu_2_(F_2_AzoBDC)_2_(dabco) SURMOF (black), after green-light-irradiation for 30 min and applying the electric field of 10 V for 15 min (blue line, 1 cycle, see Figure S4), after 4 cycles (cyan), after 14 days (dark yellow) and after 1 month (navy blue). The calculated XRD of the targeted structure is shown in gray and the in-plane XRD of the pristine sample is shown in dark wine red, see labels. The X-ray wavelength is 0.154 nm (Cu-K-alpha). **b)** Sketch of the Cu_2_(F_2_AzoBDC)_2_(dabco) SURMOF with the fluorinated azobenzene side groups that can be switched with light of 530 nm from *trans* (left) to *cis* (right) and back with 400 nm. Carbon atoms are shown in gray, oxygen in red, copper in orange, fluorine in green, and nitrogen in blue. Hydrogen atoms have been omitted.

**
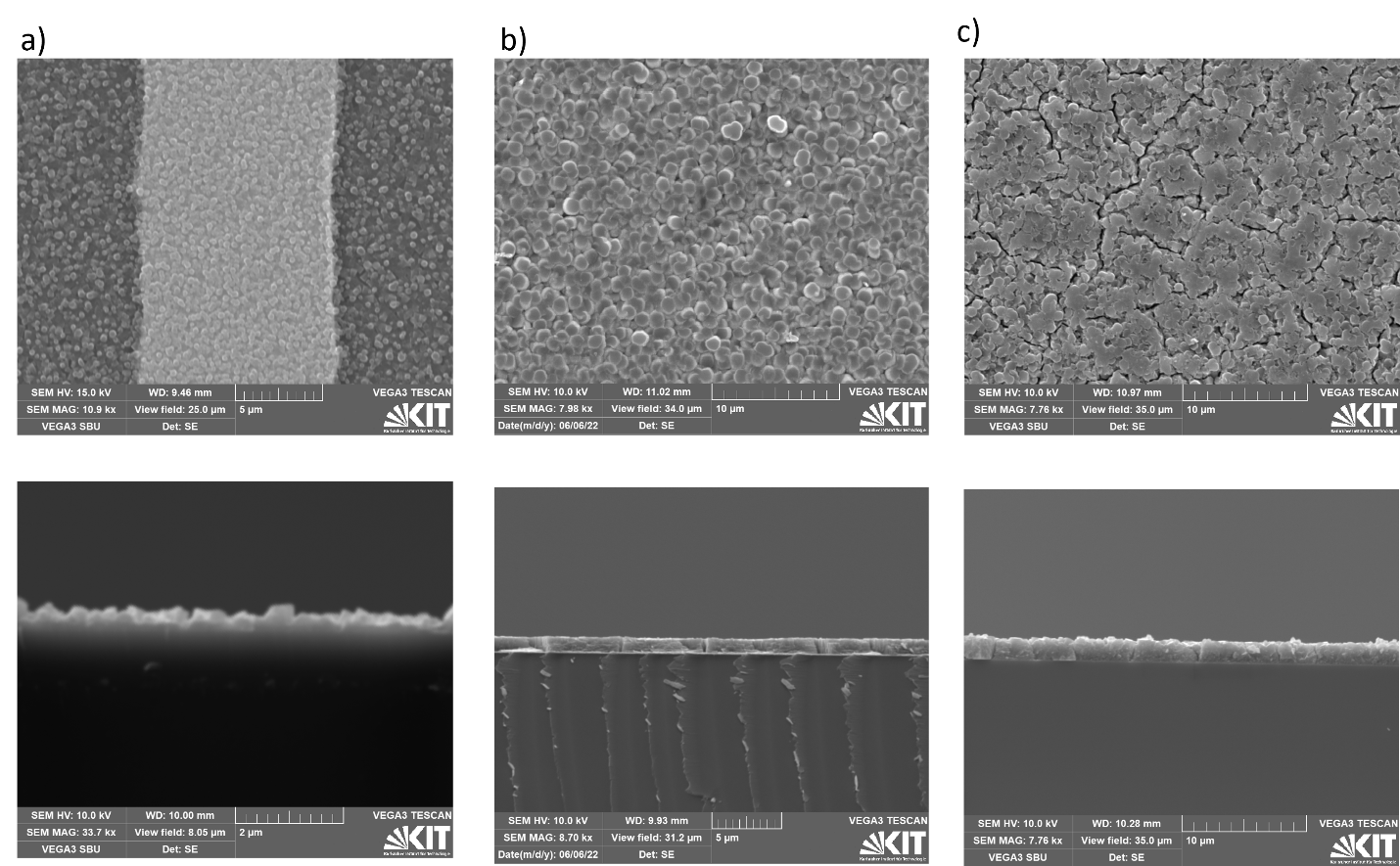
**

**Figure S2**. Scanning electron microscopy (SEM) images of the top views (top) and sideviews of a broken sample (bottom). **a)** The SURMOF was grown on a substrate with deposited interdigitated gold electrodes, which can be seen as bright stripes in the top-view image. The SURMOF thickness is ≈0.2 µm. **b)** The SURMOF was grown on a gold-coated silicon wafer. The SURMOF thickness is ≈1.2 µm. **c)** The SURMOF was grown on ITO on glass. The SURMOF thickness is ≈1.6 µm. Please note the different numbers of synthesis cycles for the sample preparation, see experimental section.

**
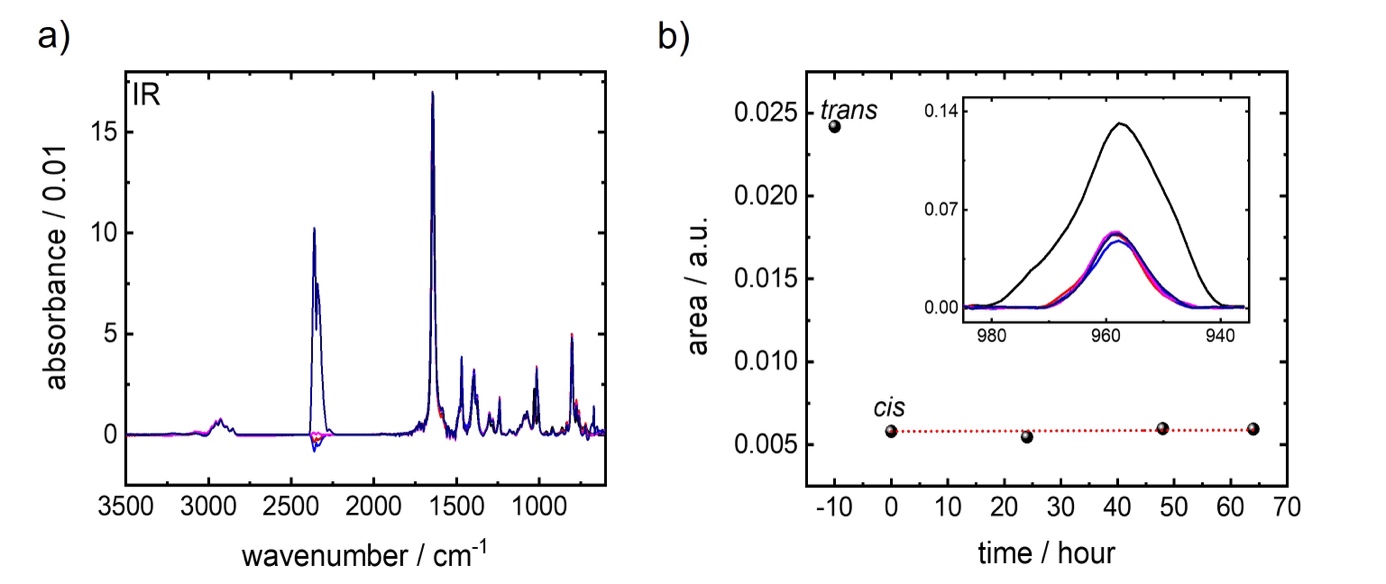
**

**Figure S3.** **a)** Infrared reflection absorption spectra (IRRAS) of the SURMOF in the thermally relaxed state (black, 100% *trans*). The spectra upon irradiation with green light (*cis*-rich state) were measured immediately after irradiation (red), after 24 h (blue), after 48 h (magenta), and after 64 h (navy blue). **b)** Area under the band at 960 cm^-1^. This band is attributed solely to *trans* azobenzene.^[1]^ The inset shows a zoom-in of the spectra in a) of the 960 cm^-1^ band. The estimated time constant for the thermal *cis*→*trans* azobenzene isomerization (in the dark without applied electric field) is 14321 h (approximately 1.6 years). The estimation is made by fitting the *cis*-rich area data points with a mono-exponential decay function, i.e. $y=A\text{⋅}$ $e^{-t/\tau}+y_{0}$. This estimation of the time constant is in line with the time constants for the thermal *cis*→*trans* isomerization of similar fluorinated azobenzene molecules in solution.^[2]^


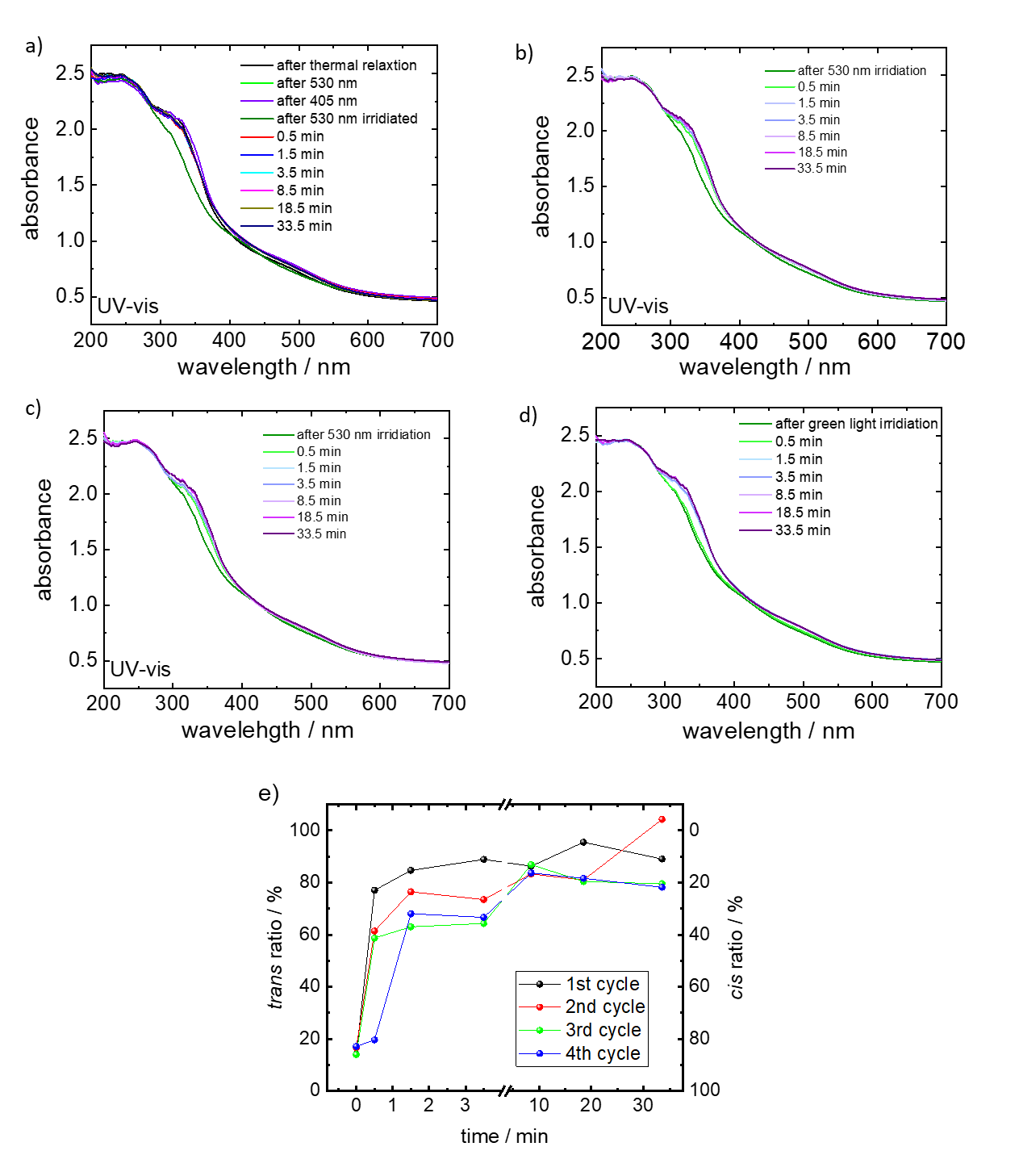


**Figure S4.** UV-vis transmission absorption spectra of the SURMOF@IDE@glass. The electric-field induced *cis*-to-*trans*-isomerization was performed for 4 cycles: **a)** first, **b)** second, **c)** third and **d)** forth cycle. Before measuring the first cycle, the sample was thermally relaxed (72 h at 80 ℃), ensuring 100% *trans* azobenzene, and irradiated with green and violet light. Then, the sample was irradiated with green light (530 nm) for 30 min. The *E*-field (1 V/µm) was applied for 0.5, 1.5, 3.5, 8.5, 18.5, 33.5 min, respectively, see labels. For the next cycle, the sample was irradiated with 530 nm again. **e)** The amount of *cis-* and *trans*-azobenzene in the SURMOF during the voltage exposure. The cycles are labelled.


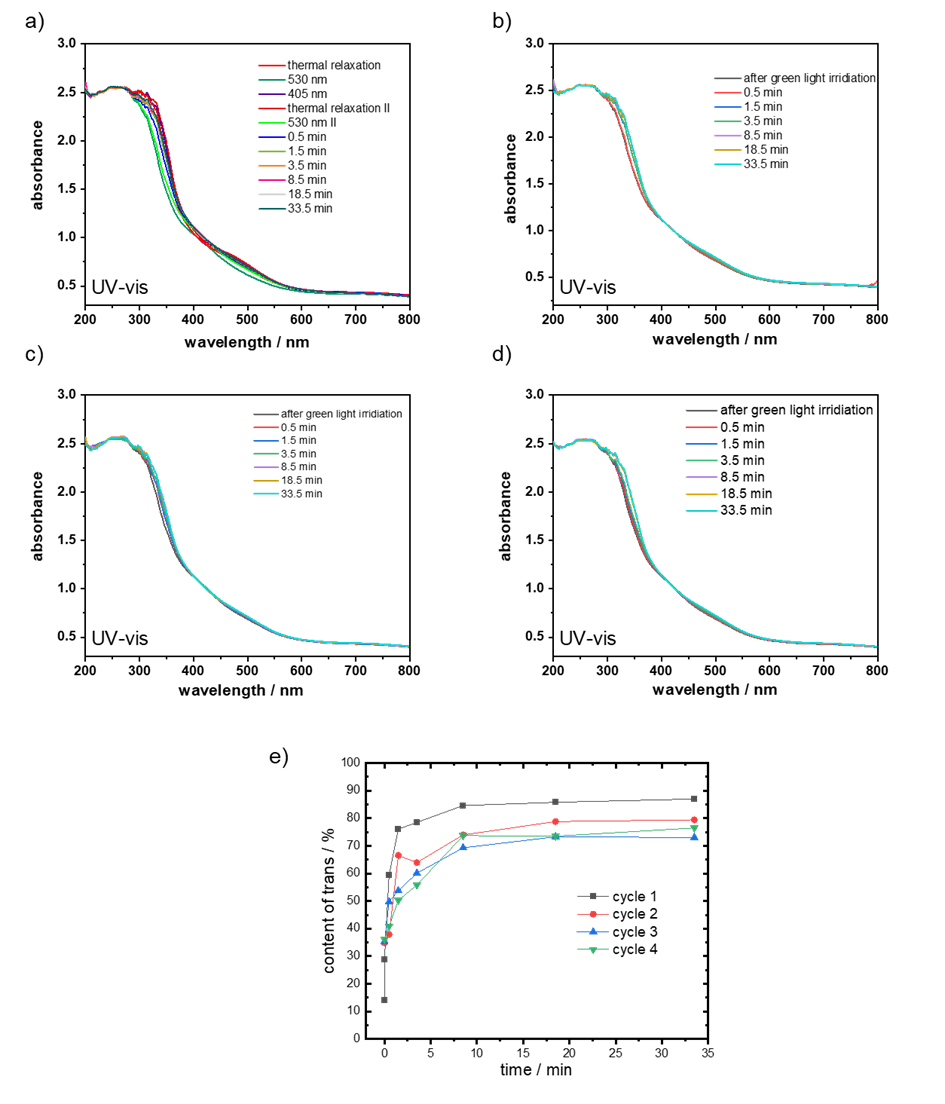


**Figure S5.** Repetition of experiments in Figure S4 with a second sample. UV-vis transmission absorption spectra of the SURMOF@IDE@glass during the electric-field induced *cis*-to-*trans*-isomerization for 4 cycles. Before measuring the first cycle UV-vis spectra, the sample was thermally relaxed (72 h at 80 ℃), ensuring 100% *trans* azobenzene. Then, the sample was irradiated with green light (530 nm) for 30 min. The *E*-field (1 V/µm) was applied for 0.5, 1.5, 3.5, 8.5, 18.5, 33.5 min, respectively, see labels. For the next cycle, the sample was irradiated with 530 nm again. **a)** first, **b)** second, **c)** third and **d)** forth cycle. **e)** The amount of *trans*-azobenzene in the SURMOF during the voltage exposure. The cycles are labelled.


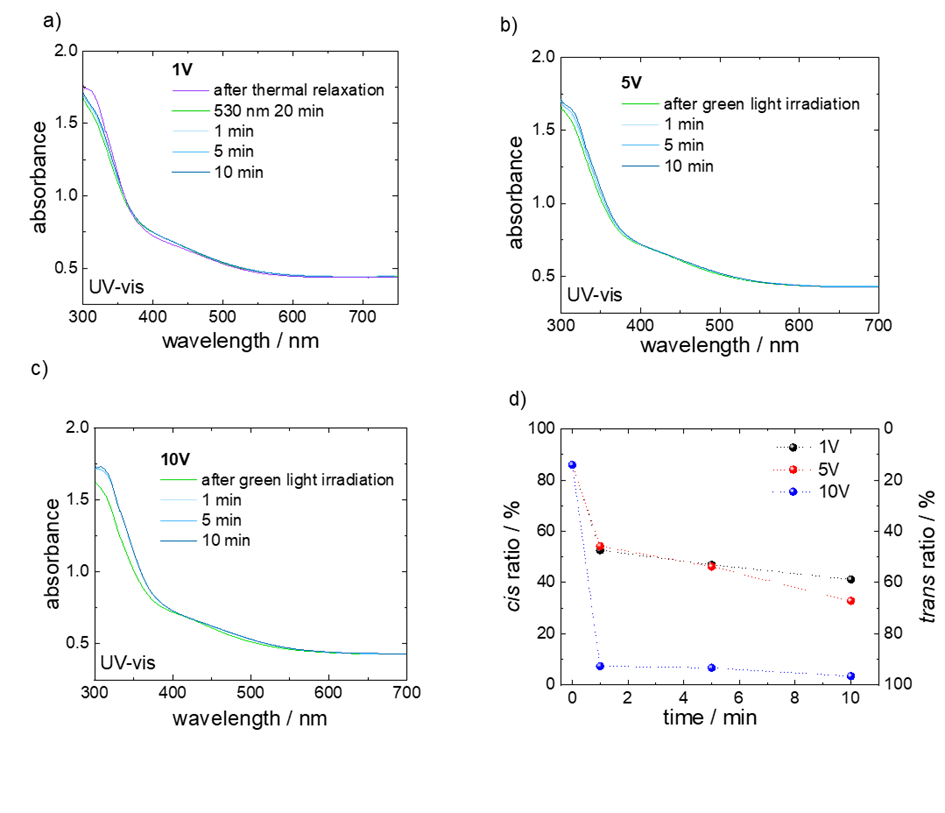


**Figure S6.** UV-vis transmission absorption spectra of the SURMOF@IDE@glass. Different voltages were applied between the IDEs: **a)** 1 V, **b)** 5 V, and **c)** 10 V. **d)** The amount of *cis-* and *trans*-azobenzene in the SURMOF during the voltage exposure. The different voltages are labelled.


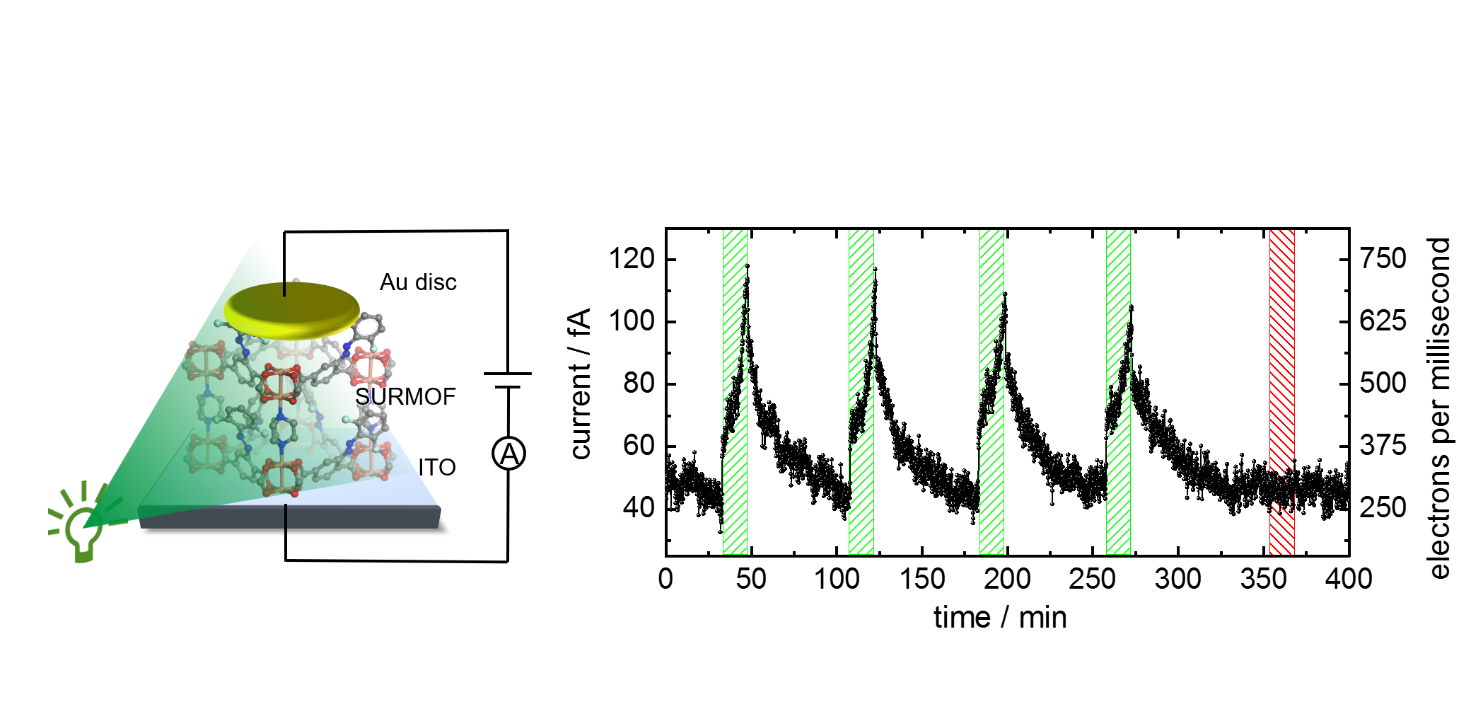


**Figure S7.** Transient current through the Cu_2_(F_2_AzoBDC)_2_(dabco) SURMOF film. The sample was irradiated four times with green light of 530 nm and once with red light of 640 nm for 15 min each, see labels. The setup with the ITO bottom electrode (on glass) and the small gold disc as top electrode is sketched on the left-hand side. The DC voltage between the top and bottom electrode is 1 V. The sample is in the *trans* state at the beginning. Before the experiment, the sample was equilibrated at a voltage of 1 V for 1 h. In comparison to the data in Figure 3, the measurements are performed with a different sample.

While an ITO-SURMOF-Au-setup, which allows the irradiation of the sample during the experiments was used in **Figures 3, 4 and S7**, an Au-SURMOF-Au-setup was used in **Figure S8**, where the sample can be irradiated only before the deposition of the Au-top-electrode, i.e. before the experiment.


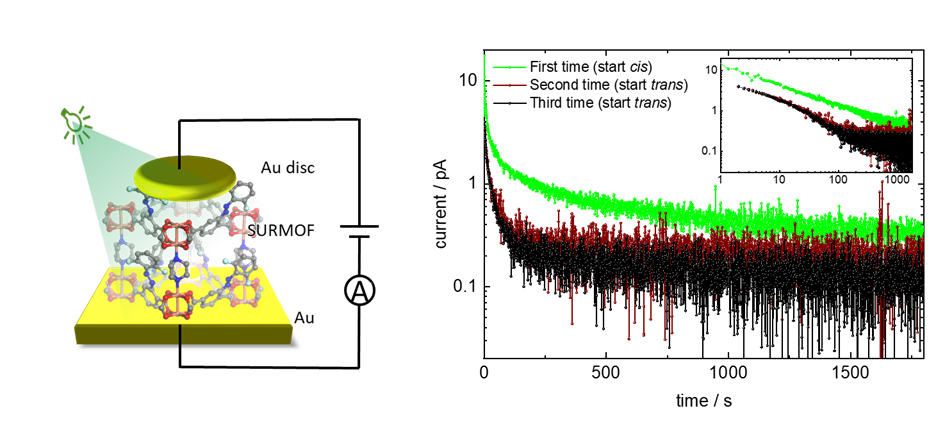


**Figure S8.** Transient current through the Cu_2_(F_2_AzoBDC)_2_(dabco) SURMOF film on gold substrate. Before the measurement and the deposition of the gold-disc top electrode, the sample was irradiated with green light, switching the sample to the *cis* state. The sample was equilibrated without voltage for 15 min to avoid any thermal effects. The 1 V DC-voltage was switched on and the current was measured for 30 min (green data) and then the sample was equilibrated in the dark for 30 min without voltage. Then, the cycle was repeated twice, wine-red and black data. For the *trans*-SURMOFs, the current is initially about 6 pA (also in Figure 6) and the current decreases with a mono-exponential time constant of about 20 s.

**
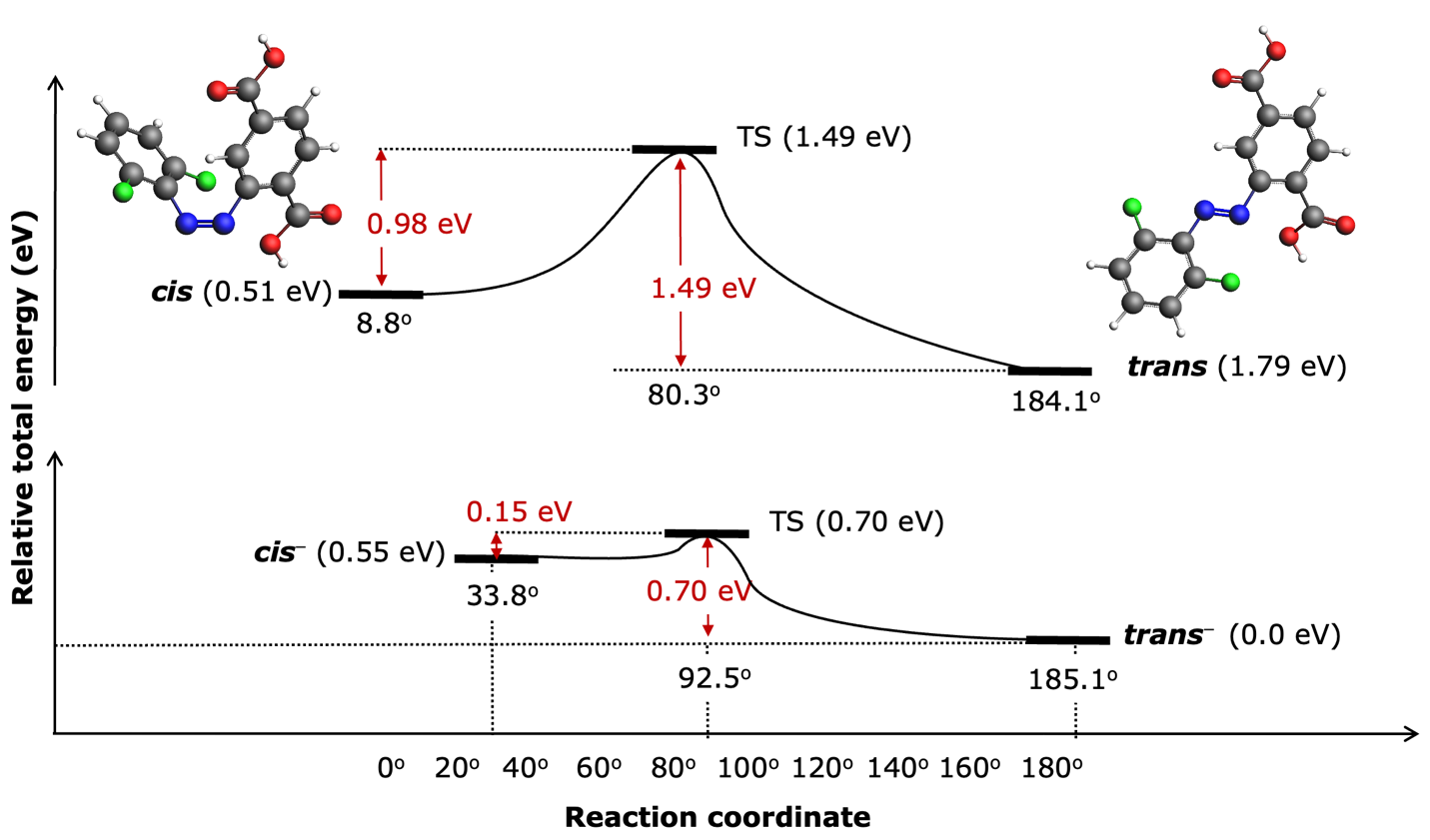
**

**Figure S9.** Energy levels of the azobenzene molecule in *trans* and *cis* form as well as of the transition state (TS) for the neutral molecule and charged with one electron. The energies for the neutral azobenzene molecule (top) as well as the corresponding radical anion (bottom) were calculated in the gas phase at the B3LYP-D3(BJ)/TZ2P level of theory with ADF2019. The x-axis is the reaction coordinate, corresponding to the C-N=N-C dihedral angle.

**
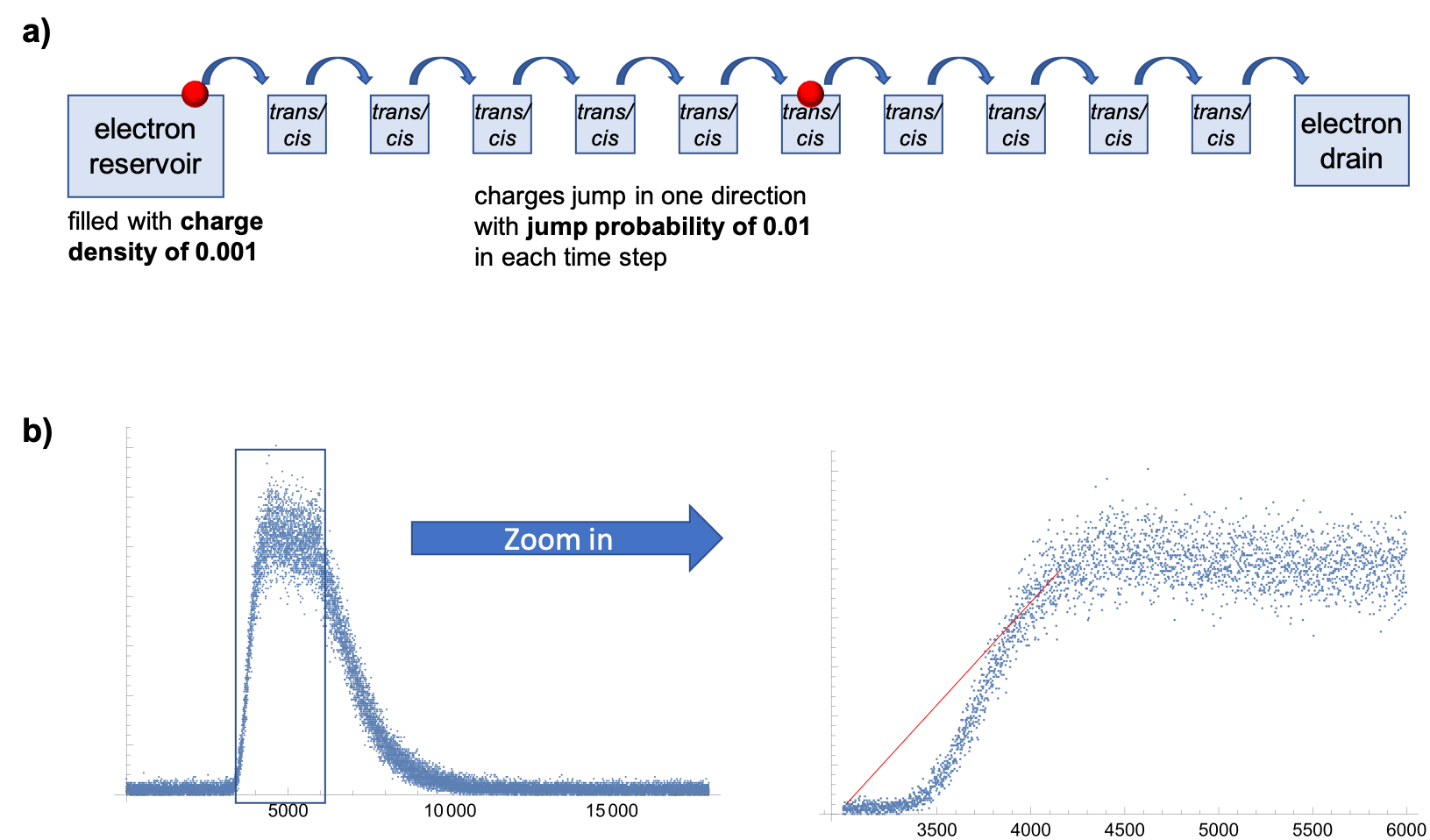
**

**Figure S10.** Monte-Carlo-simulation of the current by domino-like charge transfer and light-induced *trans-cis* pumping. **a)** Sketch of the model. The model is composed of a chain of 10 units which can be switched between two states (*trans* and *cis*, corresponding to dominoes laying or standing). This chain is in between an electron reservoir (cathode), where the charges (red spheres) are entering the chain, and a drain (anode), where the charges leave the system. Here, the electron reservoir (left) is always filled with a likeliness of 0.001. The amount of electrons at the drain (right) is recorded, corresponding to the current through the system. In the chain, the charges can only move from left to right. For each time step, each charge has a probability for such a jump of 0.01, provided the destination site contains no charge. Initially, all sites are in the *trans* state (laying dominoes). Between time-step 3000 and 6000 (highlighted as box in panel b), the states are transferred from *trans* to *cis* with a probability of 0.001 per time step, corresponding to the green-light-induced *trans*-to-*cis*-isomerization (standing dominoes). When a charge is at a site which is in *cis* state, the site changes to the *trans* state and the charge is hopping one step to the right, corresponding to the domino-flip.

**b)** The recorded current during the simulations. 400,000 chains (see panel a) have been simulated and accumulated. The main features of the experiments (Figure 3 and S6) can be found: i) The current increases when the light is switched on. ii) The current increase during the light irradiation is stronger than linear at the beginning of the irradiation (roughly between 3000 and 400 time steps here), approximately in a hyperbolic or exponential manner. iii) After a while (after roughly 4000 steps here), the current saturates. iv) The transition from the increasing current to the saturated current is rather abrupt, kink-like (not a first-order mono-exponential decay function to reach the steady-state value). v) When the light is switched off, the current decreases, approximately exponentially. To summarize, we believe it is very remarkable that the characteristics of the current kinetics are qualitatively reproduced by this simple model. The red line was added, connecting the current when the light was switched on (at 3000) with the equilibrium value was reached (at about 4000), stressing the hyperbolic current increase.

References

[1] a) K. Müller, A. Knebel, F. Zhao, D. Bléger, J. Caro, L. Heinke, Switching Thin Films of Azobenzene-Containing Metal-Organic Frameworks with Visible Light, *Chemistry – A European Journal* **2017**, *23*, 5434 – 5438; b) P. Qin, S. Okur, C. Li, A. Chandresh, D. Mutruc, S. Hecht, L. Heinke, A photoprogrammable electronic nose with switchable selectivity for VOCs using MOF films, *Chemical Science* **2021**, *12*, 15700-15709.

[2] C. Knie, M. Utecht, F. L. Zhao, H. Kulla, S. Kovalenko, A. M. Brouwer, P. Saalfrank, S. Hecht, D. Bleger, ortho-Fluoroazobenzenes: Visible Light Switches with Very Long-Lived Z Isomers, *Chemistry-a European Journal* **2014**, *20*, 16492-16501.
